# Treating primary headaches – management of migraine

**DOI:** 10.1186/1129-2377-14-S1-O1

**Published:** 2013-02-21

**Authors:** Dimos D Mitsikostas

**Affiliations:** 1 Neurology Department, Athens Naval Hospital, Greece

## 

Migraine is a common chronic neuronal disorder with episodic attacks affecting the one tenth of the general population with enormous social impact. The management of the disorder includes several approaches, both behavioral and pharmaceutical interventions, preventive or symptomatic ones. Exceptionally, invasive treatments may be required as well. A plentiful pallet of pharmacological agents covers symptomatic treatment, further divided into specific and nonspecific anti-migraine agents. Apart from triptans, ergot alkaloids are also included in the specific anti-migraine drugs. Triptans, share a common pharmacological profile targeting to 5-HT1B/1D/1F receptors with different pharmacokinetic and pharmacodynamic properties resulting to slight diverse efficacy and safety that may be important for the treatment goals. Triptans are recommended as initial treatment for moderate to severe migraine attacks, or when other agents failed to reach the expected therapeutic benefit in less-disabled patients. For migraine prophylaxis valproate, topiramate and flunarizine are recommended. In the case of chronic migraine, a disabling subform of migraine, with or without medication overuse, onabotulinumtoxinA and topiramate are suggested. A variety of behavioral treatments are available for those patients they experience increased stress in particular. Although numerous, the above treatments may not be enough to manage migraine appropriately even in the right hands, rising needs for future treatments with improved efficacy and safety profile. CGRP antagonists and specific 5-HT1F agonists are potential future symptomatic agents for migraine.

